# Draft Genome Sequence of *Bacillus simplex* DSM 1321 for Setting Up Phylogenomics in Genomic Taxonomy of the *Bacillus*-Like Bacteria

**DOI:** 10.1128/genomeA.00574-16

**Published:** 2016-06-23

**Authors:** Guo-hong Liu, Bo Liu, Jie-ping Wang, Jian-mei Che, Qian-qian Chen, Zheng Chen

**Affiliations:** Agricultural Bio-Resources Research Institute, Fujian Academy of Agricultural Sciences, Fuzhou, China

## Abstract

*Bacillus simplex* DSM 1321 is a Gram-positive, spore-forming, and aerobic bacterium. Here, we report the draft genome sequence of *B. simplex* DSM 1321, with 6,494,937 bp, which will provide useful information for setting up phylogenomics in genomic taxonomy of the *Bacillus*-like bacteria as well as for the functional gene mining and application of *B. simplex* DSM 1321.

## GENOME ANNOUNCEMENT

*Bacillus simplex* DSM 1321 is widely spread in the soil and has high similarity with *Bacillus muralis* DSM 16288^T^. With decreases in the cost of genomic sequencing, it has been proposed that whole-genome sequencing information be combined with the main phenotypic characteristics as a polyphasic approach strategy (taxonogenomics) to describe new bacterial taxa (1–4). In this study, a high-quality genome sequence of *B. simplex* DSM 1321 was sequenced, which would promote research on the genomic taxonomy of the *Bacillus*-like bacteria.

The genome of *B. simplex* DSM 1321 was sequenced with massively parallel sequencing (MPS) Illumina technology. Two DNA libraries were constructed: a paired-end library with an insert size of 500 bp and a mate-pair library with an insert size of 5 kb. The 500-bp library and the 5-kb library were sequenced using an Illumina HiSeq 2500 using a PE125 strategy. Library construction and sequencing were performed at the Beijing Novogene Bioinformatics Technology Co., Ltd. Quality control of both paired-end and mate-pair reads was performed using an in-house program. After this step, Illumina PCR adapter reads and low-quality reads were filtered. The filtered reads were assembled by SOAP*denovo* (5, 6) to generate scaffolds. All reads were used for further gap closure. Through the data assembly, 6,494,937 bp within 23 scaffolds were obtained, and the scaffold *N*_50_ was 1,760,672 bp. The average length of the scaffolds was 1,585,992 bp, and the longest and shortest scaffolds were 3,171,336 bp and 647 bp, respectively.

Gene prediction was performed on the *B. simplex* DSM 1321 genome assembly by GeneMarkS (7). Transfer RNA (tRNA) genes were predicted with tRNAscan-SE (8), ribosomal RNA (rRNA) genes were predicted with RNAmmer (9), and small RNAs (sRNAs) were predicted by BLAST against the Rfam (10) database. PHAST (11) is used for prophage prediction, and CRISPRFinder (12) is used for clustered regularly interspaced short palindromic repeat (CRISPR) identification. A total of 6,932 genes were predicted, including 6,813 coding sequences (CDSs), 5 sRNAs, 81 tRNAs, and 33 rRNA genes (14 5S rRNAs, 8 16S rRNAs, and 11 23S rRNAs). Also, 9 prophage and 3 CRISPR arrays were found in the draft genome. The average DNA G+C content was 40.11%.

### Nucleotide sequence accession numbers.

This whole-genome shotgun project has been deposited at DDBJ/EMBL/GenBank under the accession no. LWJJ00000000. The version described in this paper is version LWJJ00000000.1.
